# Classification of Fixed Point Network Dynamics from Multiple Node Timeseries Data

**DOI:** 10.3389/fninf.2017.00058

**Published:** 2017-09-20

**Authors:** David Blaszka, Elischa Sanders, Jeffrey A. Riffell, Eli Shlizerman

**Affiliations:** ^1^Department of Applied Mathematics, University of Washington Seattle, WA, United States; ^2^Department of Biology, University of Washington Seattle, WA, United States; ^3^Department of Electrical Engineering, University of Washington Seattle, WA, United States

**Keywords:** attractor networks, classification of fixed point networks, olfactory neural circuits, stimuli classification, recordings from neural population, neural dynamics, recognition of stimuli, mixed stimuli

## Abstract

Fixed point networks are dynamic networks encoding stimuli via distinct output patterns. Although, such networks are common in neural systems, their structures are typically unknown or poorly characterized. It is thereby valuable to use a supervised approach for resolving how a network encodes inputs of interest and the superposition of those inputs from sampled multiple node time series. In this paper, we show that accomplishing such a task involves finding a low-dimensional state space from supervised noisy recordings. We demonstrate that while standard methods for dimension reduction are unable to provide optimal separation of fixed points and transient trajectories approaching them, the combination of dimension reduction with selection (clustering) and optimization can successfully provide such functionality. Specifically, we propose two methods: Exclusive Threshold Reduction (ETR) and Optimal Exclusive Threshold Reduction (OETR) for finding a basis for the classification state space. We show that the classification space—constructed through the combination of dimension reduction and optimal separation—can directly facilitate recognition of stimuli, and classify complex inputs (mixtures) into similarity classes. We test our methodology on a benchmark data-set recorded from the olfactory system. We also use the benchmark to compare our results with the state-of-the-art. The comparison shows that our methods are capable to construct classification spaces and perform recognition at a significantly better rate than previously proposed approaches.

## 1. Introduction

Robust neural networked systems encode their dynamics by attractors in a low-dimensional state space. Attractors represent the response of a neural network to various inputs, as well as reflecting particular states of the system. Such networks are common in neuronal systems that process sensory stimuli, command motor systems, or store memory (Amit, 1992; Wills et al., 2005; Churchland et al., 2012). The manifestation of attractors ensures robustness of network performance, such that, for a range of network initializations and stimuli, it exhibits reliable dynamics. The simplest type of attractors are fixed points, triggered by injection of input signals (e.g., step functions) into a subset of network nodes, which after a transient response, produce a steady state pattern in output nodes. In neuronal networks, these patterns are identified as neural codes (Averbeck et al., 2006). The network is considered selective when it distinguishes between stimuli via distinct fixed points. In particular, fixed point networks produce similar fixed points for similar stimuli and distinguishable ones for distinct stimuli. The similarity is typically defined by a metric in a low-dimensional space. Such functionality is the primary principle upon which fixed point networks incorporate recognition and quantification of mixed stimuli. In addition, these networks are “robust” as they perform under high dynamic input variability, with low signal to noise ratio (SNR).

**Figure 1:**
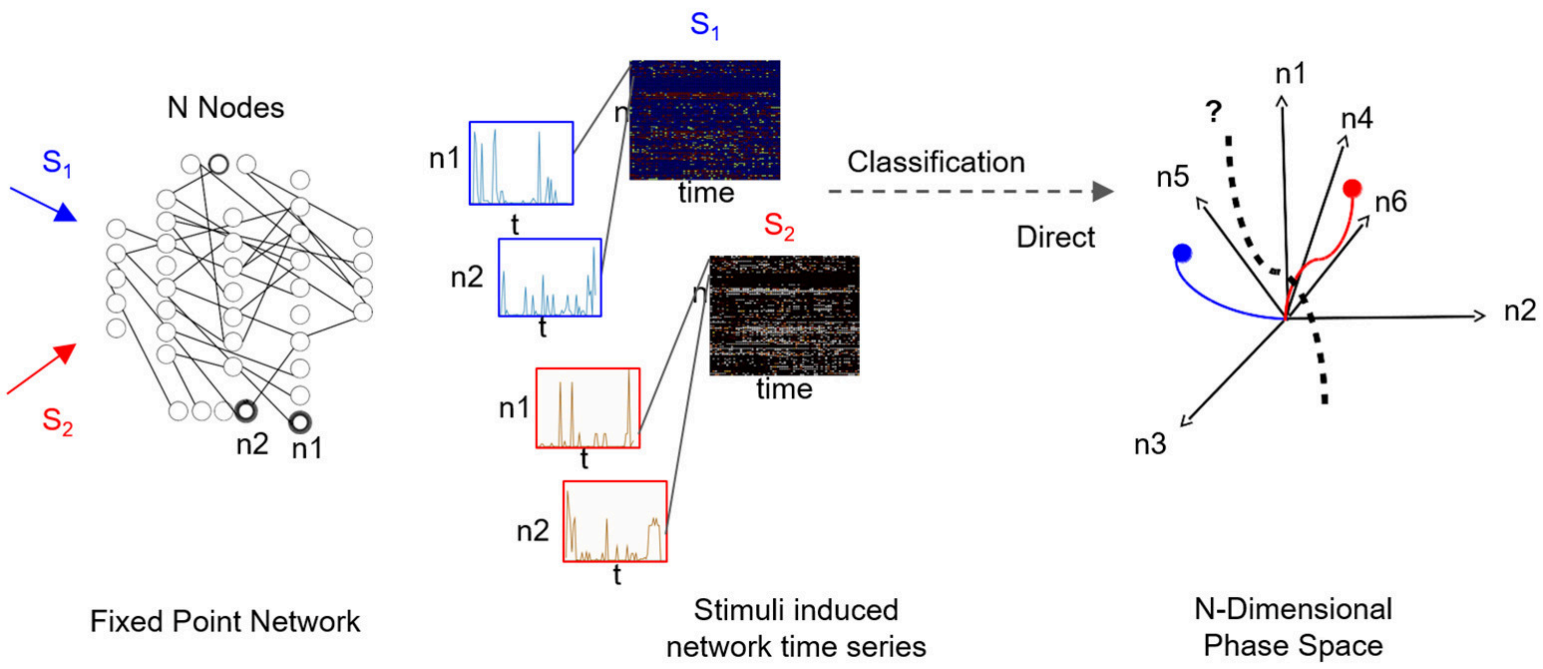
Supervised classification of fixed point network responses. Left to right: Distinct input signals, stimuli *S*_*i*_ (here *i* = 2), are injected into network's input nodes and produce fixed point dynamics in output nodes (*n*1 and *n*2 are samples of output nodes). For each stimulus, output nodes timeseries are recorded in a response matrix, with dimensions of nodes × time, where each node is a row of the matrix. Response matrices to *S*_1_ and *S*_2_ stimuli are shown as color maps and time series of sampled nodes *n*1 and *n*2 (rows 1 and 2 of each response matrix) are shown in insets. As the number of output nodes increases, finding a separating hyperplane between the dynamics represented in the nodes' space becomes complex as illustrated in the rightmost plot.

A fascinating question is how to infer the low-dimensional state space—and fixed points within it—from the network's time series data. It is particularly relevant when it is of interest to functionally characterize black-box networks, where connectivity and node dynamics are unknown, and sampling the network response to various stimuli is the only resort. Supervised sampling is often used as a structural approach for the construction of low-dimensional state space. In such an approach, distinct inputs are independently applied to the network, while network activity is being sampled (i.e., time-series of multiple node dynamics). For each input, the fragment of sampled time-series corresponds to a matrix with “nodes” and “time” dimensions, and the data-set of sampled responses corresponds to a collection of matrices (see Figure 1). The classification task for such a collection is to find the most informative low-dimensional state space where the number of distinct fixed points is the number of distinct inputs; thus the low-rank space could be used for examining the transient dynamics reaching these fixed points. Such a space would be considered optimally selective when both fixed points and associated transient dynamics with each fixed point are maximally separated (orthogonal). For example, for a collection which constitutes responses to three inputs, we expect to find three basis vectors, each representing a single stimulus.

For neuronal sensory networks, the collection would typically correspond to the instantaneous firing-rates (peri-stimulus time-histograms) of multiple neurons when the system is stimulated. We chose here to work with the olfactory neuronal network in insects, due to its particularly robust and intriguing neural coding. Within the network, the antennal lobe, the primary processing unit of olfactory information, receives input from olfactory receptor neurons and responds with output activity that discriminates between odorants, and classifies olfactory stimuli. Experiments have shown that such classification is associated with behavior, and could adapt over time or after training (Riffell et al., 2013, 2014). Furthermore, analysis of multi-unit electrophysiological recordings from output (projection) neurons shows that the network employs input induced fixed points to classify the stimuli (Mazor and Laurent, 2005).

Since the collection consists of matrices, a natural methodology for inferring low-dimensional classification space is composing the collection into a single matrix and employing classical multivariate matrix decomposition methods to identify dominant orthogonal patterns, e.g., Singular Value Decomposition (SVD), or sparse representations with L1 minimization (Sirovich, 1987; Shlizerman et al., 2012). However, in practice, due to the ambiguity in SVD (indifference for particular data structure and sensitivity to low SNR), the resulting SVD modes are mixed and unable to discriminate single inputs from the collection. Alternatively, dimension reduction could be applied on each of the matrices and produce unambiguous, low-rank representations. However, this approach introduces another problem of gathering the obtained patterns into a single, joint, meaningful representation. This is a generic problem of finding an optimal low-rank representation of multiple instances data matrix.

To address the problem, here we propose a classification method that leverages the benefits of the two approaches: orthogonality and non-ambiguity with respect to the structure. The method operates on low-rank representations obtained from individual applications of data reduction on each sub matrix and creates an orthogonal basis. Effectively, the method exclusively associates each neuron with one of the stimuli and assigns a weight for the association, and thereby called exclusive threshold reduction (ETR). We show that such an approach can successfully separate response trajectories to the various stimuli. In addition, we formulate an optimization routine, called optimal exclusive threshold reduction (OETR), which allows us to achieve maximal separation of fixed points. For evaluation and demonstration of our methodology we have obtained a benchmark database of multi-unit time series recordings from the antennal lobe projection neurons in the *Manduca sexta* moth. Neural activity was recorded as a response to stimulation with constituents of the *Datura* flower scent (single molecules and mixtures), a major food resource for the moth. The data-set is divided into a training set for construction of classification space, and a test set to evaluate our methodology and compare with other approaches. Standard and challenging tests were designed to quantify discrimination between distinct stimuli, and capturing similarity of various mixtures. For example, the methods were tested on the accuracy of classification of responses into “behavioral” or “non-behavioral” ones. Notably, since the recordings incorporate large *O*(1) variability in time and space of the input, both standard and challenging tests are significantly more difficult than in noiseless scenarios. We show that the OETR method performs most accurately and robustly on various number of dimensions of classification space and mixture inputs, out of the many methods that we have tested.

## 2. Results

### 2.1. Classification from multiple node collection

For supervised classification, we consider a set of input nodes {*I*} which receive a set of stimuli {*S*}. To monitor network response, we consider a set of output nodes denoted as {*F*}. In the training stage, *m* independent stimuli {*S*_*i*_}_*i* = 1, …, *m*_ are applied to the network, and timeseries of output nodes—that producing dynamic trajectories to the stable fixed points—are being recorded. Per each stimulus, *S*_*i*_, the recorded timeseries of output nodes correspond to a matrix [*F_S_i__*] of dimensions *N* × *T* with rows being the nodes and columns being the recording time stamps. Therefore, the training data for supervised training is the collection *C* = {[*F*_*S*_1__], …, [*F*_*S*_*i*__], …, [*F*_*S*_*m*__]}, which includes multi-node times-series of network responses (see Figure 1 for an example). The goal of the classification is to find an optimal representation such that the responses to distinct stimuli are separable. Effectively, the problem is related to finding a separating hyperplane between each fixed point and its associated trajectories that approach/leave it and all other fixed points and their associated trajectories.

**Figure 2:**
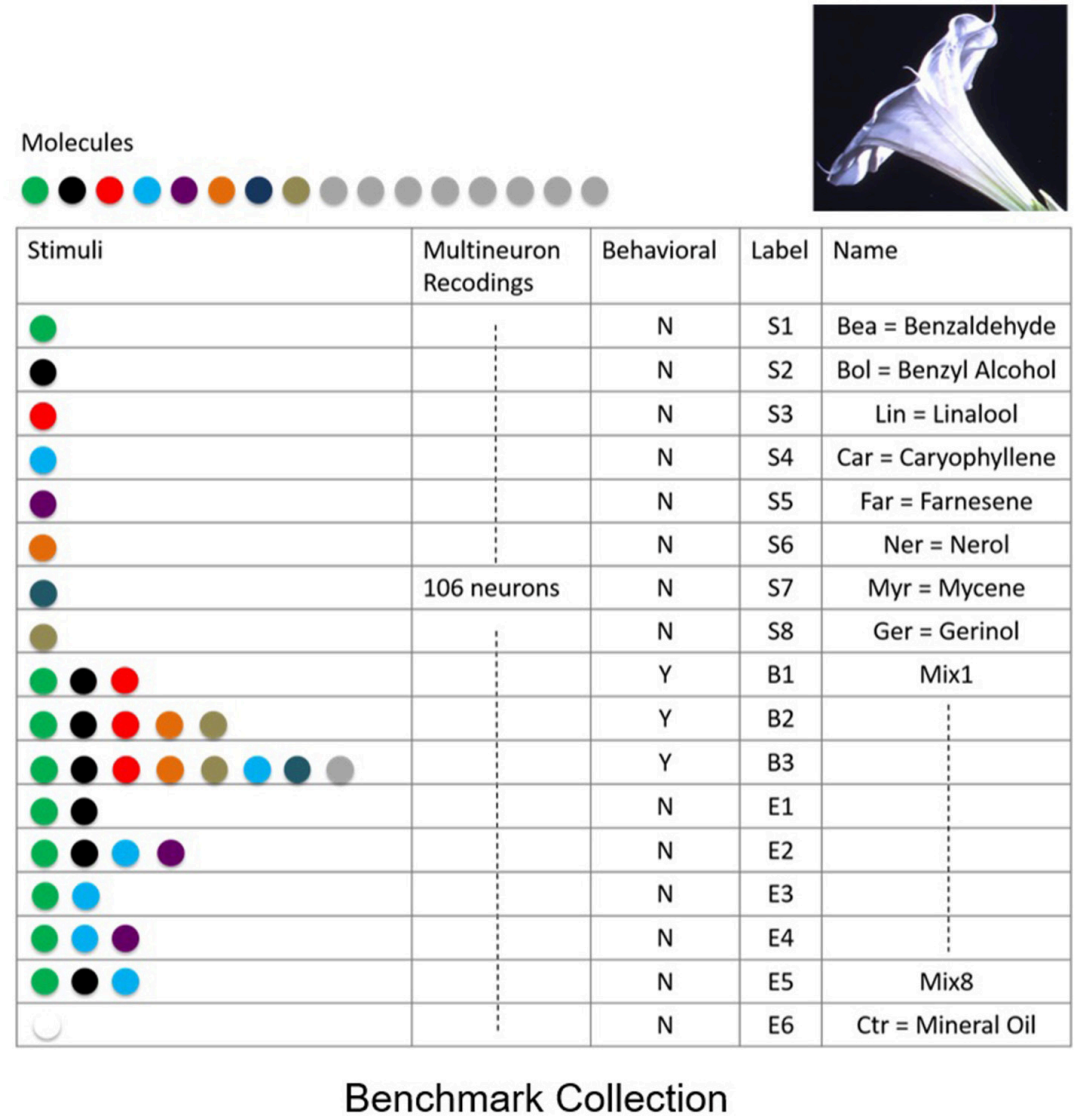
Benchmark data set of supervised recordings and superpositions of stimuli from the olfactory network. Recordings of extracellular neural responses of 106 neurons for 8 mono-molecular stimuli (odorants) that constitute the Datura scent, labeled as S1,…,S8. In addition, responses to mixed stimuli labeled as behavioral B1,…,B3, non-behavioral E1,…,E5 and control stimulus E6 were recorded. Responses were recorded over 5 distinct trials for each stimulus.

To quantify the success of various classification methods we established a benchmark data-set for classification and recognition. The benchmark consists of a collection of neural responses of a neurobiological network that exhibits fixed point dynamics. Specifically, to create the benchmark, we have obtained electrophysiological multi-neuron recordings from projection (output) neurons within the antennal lobe neuronal network, the primary olfactory processing unit in insects. It has been shown that the response of the network to odor stimuli is expressed in terms of fixed point dynamics of projection neurons (Mazor and Laurent, 2005). To obtain the benchmark, spiking activity of multiple projection neurons (*N* = 106) was recorded from the antennal lobe of *M. sexta* moth subject to distinct odor molecules (odorants) and their mixtures extracted from the *Datura wrightii* flower, a major food resource for the moth. Since realistic response times are of the order of few hundreds of milliseconds (200–400 ms), each response was recorded for 1 s. The recordings were repeated over 5 trials with 2-min intervals in between the trials. The benchmark includes responses to 8 distinct odor molecules [labeled as Bea (S1), Bol (S2), Lin (S3), Car (S4), Ner (S5), Far (S6), Myr (S7), Ger (S8)], 1 control odor (labeled as Ctr), and 8 mixture odors (labeled as B1, B2, B3, E1, E2, E3, E4, E5, E6), see table in Figure 2. Mixtures are labeled as “behavioral”—to denote that the behavioral response to these stimuli is similar to the response to *Datura* floral scent, or “non-behavioral”—to denote the mixtures and odorants which do not elicit a significant behavioral response. To work with continuous time series, neural spiking activity was transformed to nonnegative activity representing peri-stimulus time histograms (instantaneous firing rates).

**Figure 3:**
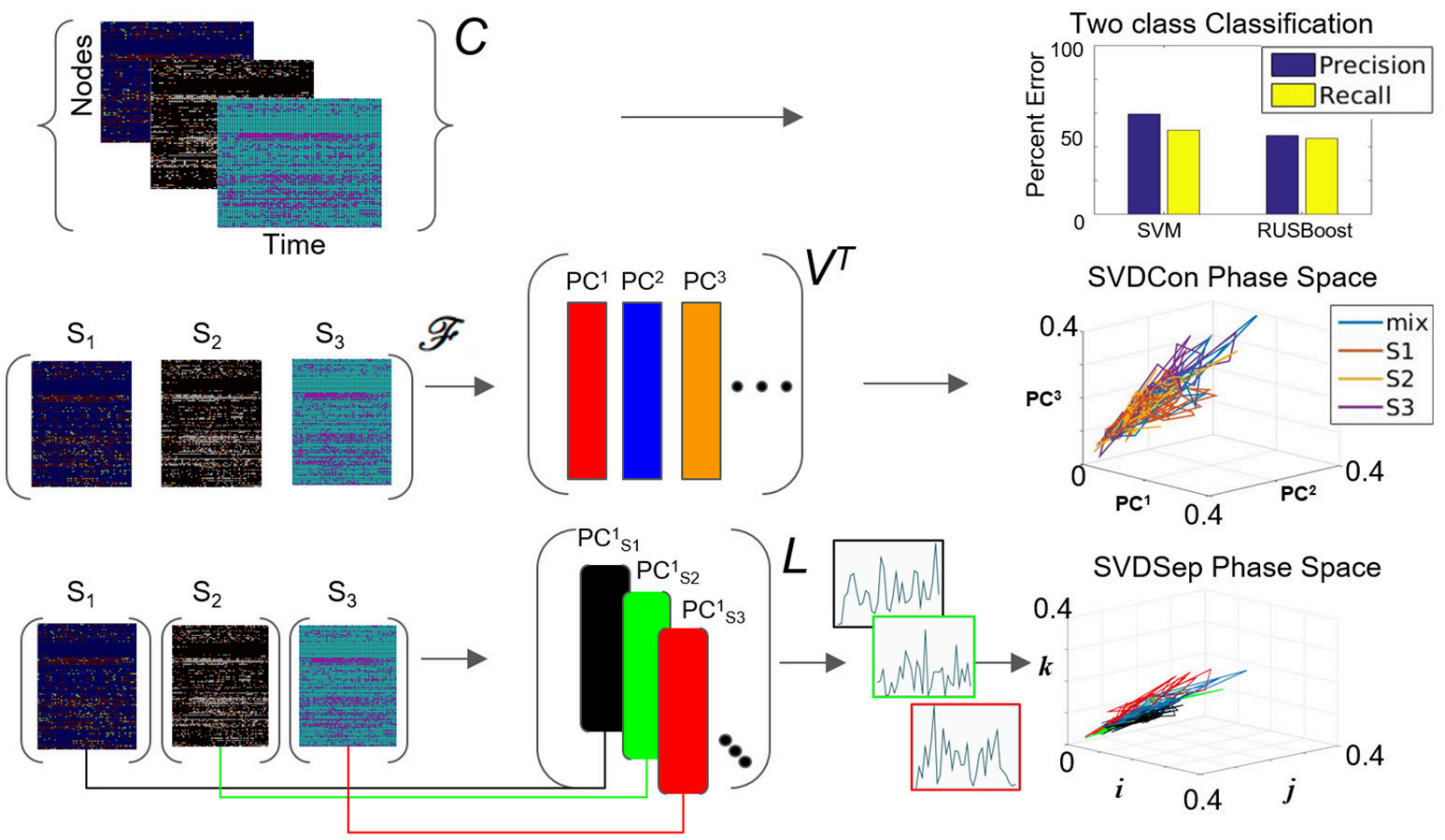
Possible methods for classification from multiple node timeseries collection. Top row: Application of SVM and RUSBoosting classifiers to perform binary classification directly on the collection *C* in the benchmark set. Precision and recall percentages are computed and shown in the right graph. Middle row: SVDCon method—Treatment of the collection as a concatenated matrix, Equation (1). Projection of the responses onto low-dimensional space spanned by *PC*^1^−*PC*^3^ obtained from SVD on concatenated matrix shown on the right. Bottom row: SVDSep method—Treatment of the collection as a set of individual matrices, Equation (3) and projection onto the first PC vector of each matrix SVD, shown as trajectories in Cartesian space spanned by unit vectors (i,j,k) on the right.

With the established benchmark we first demonstrate that finding hyperplanes separating the data points is not obvious, due to the high-dimensionality and variability of the data (noise). To test several standard methods with the benchmark, we first used the Support Vector Machine (SVM) classifier (Bishop, 2007; Murphy, 2012) with a binary classification scheme for which the data was separated into two classes: responses triggered by stimuli of interest and responses triggered by other stimuli. SVM is supposed to classify the responses by finding the optimal hyperplane separating all data points into one of the two classes (see further details on the method in section 4). For this binary classification problem we found the performance to be low, with an average classification accuracy of approximately 50% for various binary classes, as we show in Figure 3. We find that both precision (percentage of true positives out of instances classified as positive) and recall (percentage of true positives out of instances expected to be positive) errors are ≈60% and ≈50% respectively, indicating poor performance in the usefulness of the classification (precision) and completeness of classification (recall), comparable to a random guessing strategy for the binary test sets that we have created.

A possible explanation for the failure of SVM on the benchmark is that the points are linearly inseparable. We, therefore, tested nonlinear versions of the classifier, e.g., by using a Gaussian kernel, however, they produced similar classification errors. Another hypothesis for the low performance of SVM could stem from response dynamics that reside in a lower dimension than both the nodal dimension and the number of data points upon which the separating hyperplane is computed. This creates a situation where the data is being overfitted. Another obstacle could be in the form of the classes being imbalanced, i.e., when SVM classifiers are trained on an imbalanced data-set, they can produce biases toward the majority class and misrepresent the minority class. We, therefore, tested other techniques designed to deal with class imbalances, such as SMOTING and RUSBoost (Seiffert et al., 2010) with the benchmark. However, we did not obtain significant improvement with these methods compared to SVM (Figure 3). In conclusion, we observe that direct classification from the complete data-set is difficult, and methods that incorporate pre-processing of the data are necessary. In particular, we identify that determining an appropriate low dimensional state space, where distinct fixed points and their associated dynamics are easily separable, could significantly simplify classification.

### 2.2. Classification using matrix decomposition applied to timeseries collection

Since classification methods applied to the high-dimensional collection *C* are unable to provide robust representation, an alternative approach is to represent the data in low-dimensional space. The structure of the collection *C* as a set of matrices suggests that matrix decomposition could be a useful tool for finding such low-dimensional basis. There are several possibilities to create a matrix to be used for decomposition from the collection *C*. The first possibility is to format the collection into a concatenated matrix consisting of sub-matrices *F*_*S*_*i*__

(1) $$\begin{array}{l} C=\{[F_{S_{1}}],[F_{S_{2}}],[F_{S_{3}}],\ldots \}\to ℱ=[F_{S_{1}},F_{S_{2}},F_{S_{3}},\ldots ]. \end{array}$$

Data reduction methods can be then applied to the matrix F to decompose it into vectors ${\vec{a}}_{k}\left(t_{i}\right)$ (time-dependent coefficients) and ${\vec{g}}_{k}\left(j\right)$ (spatial patterns/neural codes):

(2) $$\begin{array}{l} ℱ(t_{i},j)=\sum_{k=1}^{n}{\vec{a}}_{k}(t_{i}){\vec{g}}_{k}(j). \end{array}$$

where *t*_*i*_ denotes the times at which responses where recorded and *j* denotes indices of the nodes from which responses where recorded.

Our goal is to find the most informative decomposition where the number of patterns is equal to the number of distinct inputs, and these patterns span a low-rank space of projections where the dynamics, i.e., time-dependent coefficients ${\vec{a}}_{k}\left(t_{i}\right)$, and their superpositions can be examined. For example, for a matrix which constitutes responses to three inputs, we expect to get three pattern vectors, each representing a single stimulus. Classical multivariate decomposition of the matrix F, e.g., Singular Value Decomposition (SVD)/Principal Component Analysis (PCA) that decompose the matrix F into *UΣV*^*T*^, or sparse representations computed by L1 minimization, can identify dominant orthogonal patterns (Golub and Van Loan, 2012; Shlizerman et al., 2012).

When computing the SVD for the matrix F, the procedure we call SVDCon, the column vectors of orthonormal matrix *V*^*T*^ correspond to pattern vectors. The *k*-th column vector of *V*^*T*^, the vector ${\vec{g}}_{k}\left(j\right)$ (or denoted as *PC*^*k*^ in Figure 3), is the *k*-th pattern. Each pattern has associated time-dependent coefficients vector ${\vec{a}}_{k}\left(t_{i}\right)=\sigma_{k}{\vec{\alpha }}_{k}\left(t_{i}\right)$, where ${\vec{\alpha }}_{k}\left(t_{i}\right)$ is the *k*-th row vector of the orthonormal matrix *U* and σ_*k*_ is the *k*-th singular value, the *k*-th element of the diagonal matrix Σ. Singular values indicate the weight of each pattern vector, since they are non-negative elements ordered as σ_1_ ≥ σ_2_ ≥ ⋯ ≥ 0 and since both ${\vec{\alpha }}_{k}\left(t_{i}\right)$ and ${\vec{g}}_{k}\left(j\right)$ are normal vectors. To determine the relative weights of these pattern vectors it is possible to define the relative energy *H*_*k*_ of each pattern, such that $H_{k}=\sigma_{k}^{2}/\overset{n}{\underset{i=1}{\sum }}\sigma_{k}^{2}$ which yields that the total energy is normalized $\overset{n}{\underset{k=i}{\sum }}H_{i}=1$, and each *H*_*k*_ indicates the relative “percentage” of the energy in the pattern *PC*^*k*^. The distribution of *H*_*k*_ values provides an estimate of the effectiveness of the decomposition to find a low dimensional representation. When several first *H*_*k*_ values stand out, it identifies the patterns corresponding to these singular values are more dominant than others and the truncation of the remainder modes would maintain a reasonable approximation of the original matrix.

We apply SVDCon onto a concatenated matrix F generated from three response matrices, $F=\left[F_{S_{1}},F_{S_{2}},F_{S_{3}}\right]$ to three distinct odorant stimuli from the benchmark set. We observe that the distribution of *H*_*k*_ values is such that only the first singular value stands out (*H*_1_ = 0.5) and all others are significantly smaller. Such a distribution typically indicates that SVD is unable to capture the variability in the data. Furthermore, examination of time dependent coefficients associated with the first three patterns show that they are overlapping and indistinguishable (Figure 3 middle row). Effectively, these results indicate that there is a discrepancy between the expected three-dimensional state-space and the outcome of SVDCon.

Another way to format the collection is to consider each response matrix *F*_*S*_*i*__ separately, i.e.,

(3) $$\begin{array}{l} C=\{[F_{S_{1}}],[F_{S_{2}}],[F_{S_{3}}],\ldots \}\to [F_{S_{1}}],[F_{S_{2}}],[F_{S_{3}}],\ldots \end{array}$$

Using this form, SVD can be applied to each matrix; a procedure we call SVDSep. In this procedure, *m* sets of decompositions are obtained, where *m* is the number of distinct stimuli. In Figure 3, we apply SVDSep onto three stimuli collection, as for SVDCon, however, here it is formatted into three separate response matrices [*F*_*S*_1__], [*F*_*S*_2__], [*F*_*S*_3__], and obtain three decompositions. We observe that each decomposition is dominated by 1 pattern (*H*_1_ > 0.5 for each of the three matrices).

Therefore, collecting dominant pattern vectors from all decompositions into a common set could represent a projection space for the distinct responses. More generally, a procedure following SVDSep would take the first pattern vector $PC_{S_{i}}^{1}$ (i.e., most dominant mode) from each decomposition of [*F*_*S*_*i*__] and store them as column vectors in a library matrix *L*, which has the form:

$$\begin{array}{l} L=\left[\begin{array}{ccc} | & | & \;\\ PC_{S_{1}}^{1} & PC_{S_{2}}^{1} & \cdots \\ | & | & \; \end{array}\right] \end{array}$$

where there are *n* rows accounting for every node measured in the network. Notably, column vectors of *L* are taken from separate decompositions and therefore non-orthogonal to each other and do not share the same Euclidean space. Therefore, projections onto the column vectors of the library *L*, shown in Figure 3 bottom row as points in standard Eucledian space (spanned by Cartesian unit vectors i,j,k), are not guaranteed to be non-overlapping. Indeed, we observe that that projection of the response matrices onto *L* for the constructed library *L* from the benchmark experiment of three distinct odorants, does not produce separable fixed points and associated trajectories (see Figure 3 bottom row). The non-orthogonality of the basis warrants development of an approach to transform the library so that the projected fixed points and their trajectories are maximally separable.

### 2.3. Optimal exclusive threshold reduction method

**Figure 4:**
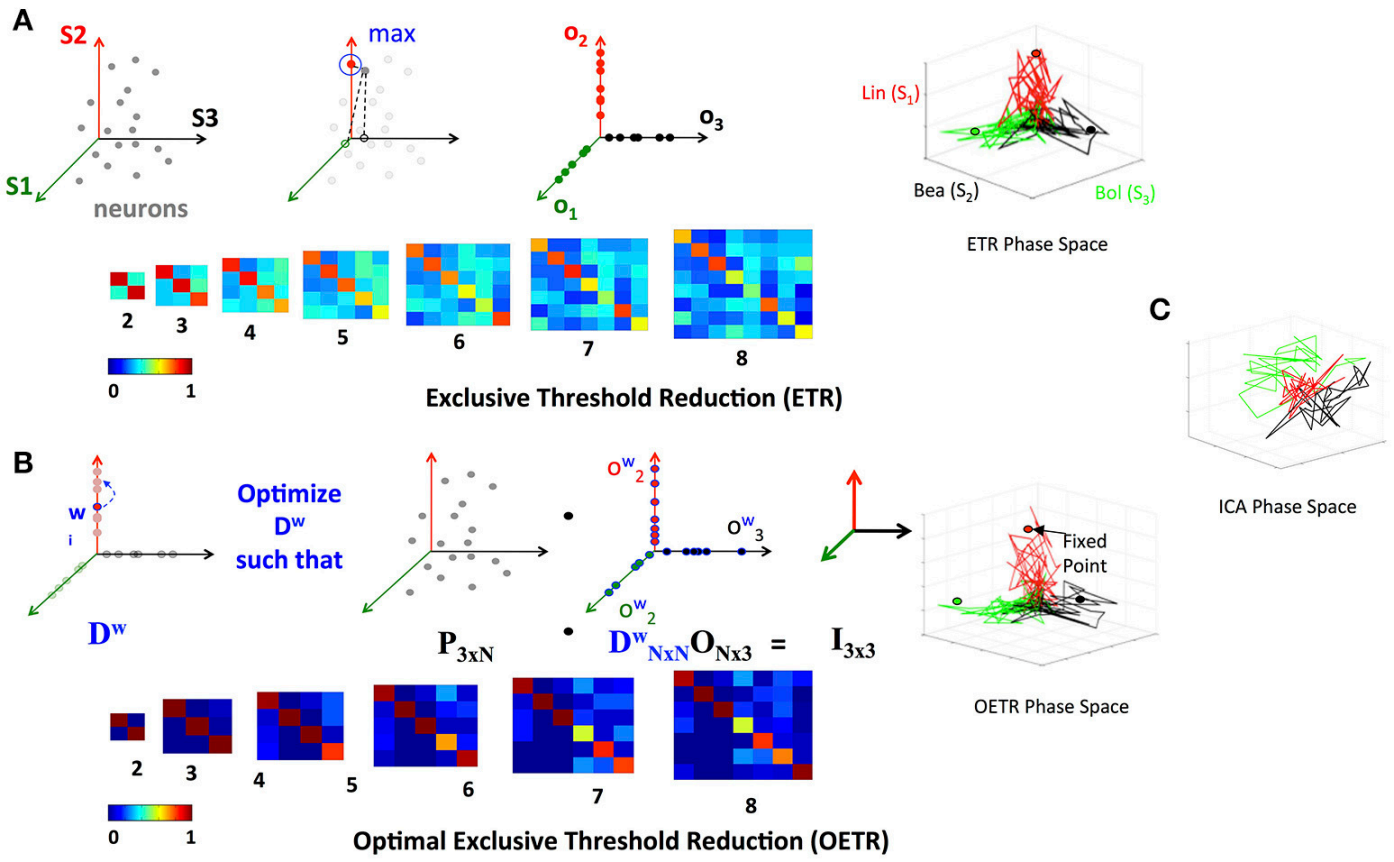
Exclusive Threshold Reduction (ETR) and Optimization (OETR). **(A)** The exclusive threshold reduction (ETR) method applies a maximizing rule (top left) to each neuron/node in the matrix *L* and produces the matrix *O* where *L*^*T*^*O* defines coordinates of fixed points (bottom left). Projection of distinct stimuli trajectories onto the matrix *O* resulting in a phase space where each stimulus trajectory is correlated with its associated axis (top right; compare with **C**). **(B)** Optimal exclusive threshold reduction (OETR) re-weights the nodes by solving a convex optimization problem, where *D*^*w*^ is optimized to find the optimal weighting of *L*^*T*^*D*^*w*^*O* = *I* (top left). The coordinates of fixed points are then defined as *L*^*T*^*D*^*w*^*O* (bottom left). This further separates the transient trajectories and associated fixed points (top right). **(C)** Projection onto basis obtained by ICA method.

To find the separation between fixed points associated with distinct stimuli, we propose a simple method that will obtain an orthogonal set of vectors from the matrix *L*. The method, called exclusive threshold reduction (ETR), operates on the nodes dimension of *L* (rows): it selects the maximal value in each row and sets to zero all other elements in that row. The procedure is performed on each row, and, once completed, produces a new matrix with *N* non-zero elements out of *N*^2^ elements. Effectively, ETR is associating each node to a stimulus. For example, if for node *n*_*i*_ the element in the third column is the maximal element, then node *n*_*i*_ is associated with *S*_3_ and the weight for the association is the value of the element, see the top row of Figure 4 for graphical illustration of the method. The ETR method could be summarized as a procedure which performs clustering of nodes into stimuli clusters. Each stimulus cluster is an axis in Figure 4. The ETR method attempts to associate each node with an axis and to assign a weight for the association. By definition, ETR guarantees an orthogonal set of vectors, i.e., an orthogonal matrix *O* of the form 
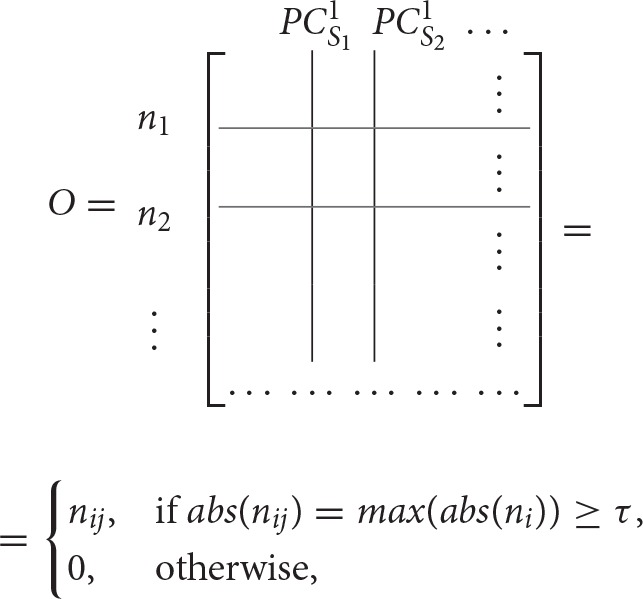
 where τ is the threshold value. If τ = 0, as in many applications, the generated matrix *O* is a basis for the nodes space and a projection space for trajectories associated with stimuli. The matrix defines a mapping from the high- to low-dimensional system. Such a mapping was used to recover network connectivity in conjunction with the Proper Orthogonal Decomposition of population model equations for the antennal lobe neuronal network (Shlizerman et al., 2014). The mapping is supposed to group node responses and capture the exclusive features associated with each stimulus. When we test the projection of three odorant benchmark matrices onto the matrix *O*, producing time-dependent trajectories in stimulus space, we observe that the trajectories are separate from each other. We also locate the fixed points, which coordinates are defined as the outcome of the product *L*^*T*^*O*. We find their location to be close to the stimulus axis of each trajectory (Figure 4A). These experiments indicate that the ETR method is effective in mapping distinct trajectories to their own axes, and can facilitate a distinct classification space.

To test the scalability of the approach we apply ETR on various subsets of responses to odorants in the benchmark set. We start with two odorants and increment by one the number of included matrices to eight odorants. Application of ETR on the latter produces 8-dimensional space (eight column matrix *O*). In these experiments, we further confirm the scalable performance of the approach and its ability to produce separate fixed points and trajectories (as shown in Figure 4A).

While ETR turns out to be valuable in separating trajectories, it does not ensure that the fixed points are optimally separated, especially when the number of matrices in the collection grows. To optimally separate the fixed points, we consider additional weights contained in the diagonal matrix *D*

(4) $$\begin{array}{l} D^{w}=\left[\begin{array}{cccc} w_{1,1} & 0 & \ldots & 0\\ 0 & w_{2,2} & \ldots & 0\\ \vdots & \vdots & \ddots & \vdots \\ 0 & 0 & \ldots & w_{n,n} \end{array}\right]. \end{array}$$

The formulation assigns weights that scale the weight of each individual node obtained by ETR to ensure that the fixed points are orthonormal (Figure 4B) and thus satisfies the assumption of distinct stimuli used in supervised classification. With such scaling, the coordinates of the fixed points are computed as *L*^*T*^*D*^*w*^*O* and in order for them to be orthonormal are expected to be exclusive in the associated axis of each stimulus. We therefore require that the coordinates of the fixed points are represented by the identity matrix, i.e., *L*^*T*^*D*^*w*^*O* = *I*. To solve for *D*^*w*^, we formulate the following convex optimization problem

(5) $$\begin{array}{l} \underset{D^{w}}{\min}\|L^{T}D^{w}O-I{\|}_{Fr}. \end{array}$$

We denote the generalized approach of solving the optimization problem above, in conjunction with the ETR method, as Optimal Exclusive Threshold Reduction (OETR) method. It is expected to produce optimally separable projected trajectories for multiple distinct stimuli and hence the vectors of *D*^*w*^*O* are optimal axes of the classification state space. To solve the optimization problem, computational packages for convex optimization can be used. For example, we use the *CVX* package implemented in MATLAB (Grant et al., 2008). Application of OETR to the three distinct stimuli benchmark set indeed produces more optimally separable fixed points and their associated trajectories as shown in Figure 4B.

Next, we compare OETR with other approaches, such as the Independent Component Analysis (ICA), to exclude that the benchmark set is too easily separated. The ICA method that we apply is an information-based algorithm (Infomax) particularly designed to obtain separable signals from a collection of inseparable signals, such as the library *L*, see section 4 for more details on our ICA implementation and Refs (Hyvärinen, 1999; Hyvärinen and Oja, 2000; Langlois et al., 2010). Our results show that projections on the ICA vectors remain overlapping and do not produce efficient classification (Figure 4C).

### 2.4. Recognition metrics and classifiers based on the classification state space

With the classification space constructed using ETR and OETR, we consider its utilization for recognition and classification of novel stimuli. These stimuli include mixtures of odorants, and mono-molecular odorants, upon which the axes of the space were constructed. Notably, the responses to all stimuli are examined on a trial by trial basis. Thereby, in the case of responses to mono-molecular odorants, each trial is a novel stochastic realization of the trajectory relative to the one used for space construction.

Since the fixed points and their associated trajectories are expected to be well separated in the classification space, we propose the convex hull metric, defined by the fixed point and its associated trajectory, as a classifier. A simplification of the convex hull (and more robust) is an *m*-dimensional hyperellipse centered at the fixed point

(6) $$\begin{array}{l} \begin{array}{l} q={\left(\frac{x_{1}-c_{1}}{r_{1}}\right)}^{2}+{\left(\frac{x_{2}-c_{2}}{r_{2}}\right)}^{2}+\ldots +{\left(\frac{x_{m}-c_{m}}{r_{m}}\right)}^{2}-1,\\ s_{t}=\left\{\begin{array}{cc} 1 & \text{for }q\leq 0\\ 0 & \text{for }q>0 \end{array}\right\}, \end{array} \end{array}$$

where *m* is the dimension of the classification state space. The metric *s*_*t*_ is a pointwise metric, which provides for each point of the tested projected trajectory *x*_1_(*t*), …, *x*_*m*_(*t*), sampled at time *t*, whether it lies inside the hyperellipse (*q* ≤ 0 : *s* = 1) or outside (*q* > 0 : *s* = 0). Integration of the pointwise metric over the time of the response [for the benchmark here it is the time of the stimulus being ON (500 ms)] provides a total score *S* of the trajectory being located within the hyperellipse

$$\begin{array}{l} Rec=\frac{S}{T},\;\;\;S=\sum_{t=t_{0}}^{T}s_{t}. \end{array}$$

**Figure 5:**
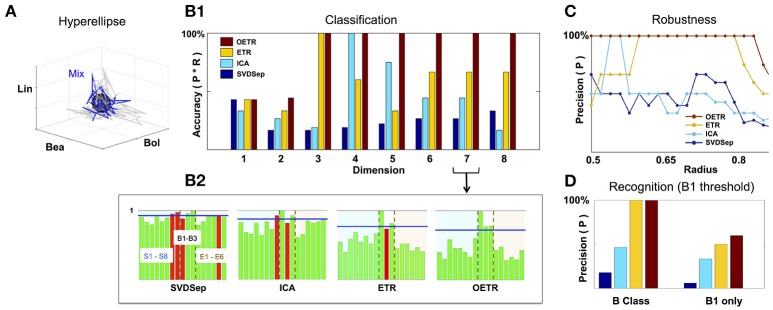
Classification and recognition employing the classification space. **(A)** ETR projection of response that corresponds to B1 (blue) and its associated 3D hyperellipse (black sphere) that is used for recognition of B1. All points that fall inside the hyperellipse are marked as “recognition evidence” (*s*_*t*_ = 1). For reference, we also show the projections of responses to B1 constituents, S1, S2, S3, in gray. **(B**_1_**)** Classification accuracy into behavioral/non-behavioral classes for OETR (red), ETR (yellow), ICA (light blue), SVDSep (navy) for increasing dimension of the Classification Space. **(B**_2_**)** Breakdown of normalized 〈*Rec*〉 scores per per stimulus (for 17 stimuli) for each method and *m* = 7. The horizontal blue line indicates the threshold line *d* for identification whether the response is similar to B1/behavioral. The bars within the dashed lines correspond to behavioral stimuli (B1–B3). Other bars correspond to non-behavioral. The color of the bars symbolize correctness of classification: correctly classified stimuli are marked by green, and incorrectly classified stimuli are marked by red. **(C)** Precision of each method when the radius of the classifying hyperellipse (sphere) is varied. **(D)** Recognition precision using B1 sphere to recognize B1 responses (right) and when false positives from behavioral **(B)** class are allowed (left).

The score *S* indicates the similarity of the tested trajectory with the stimulus trajectory. Normalization of *S* over the total number points provides a recognition ratio score *Rec*. Such a metric is simple to implement and is designed to harness the optimal separation of fixed points in the classification space. Computation of *Rec* is expected to provide consistent scores for testing similarity between trajectories in the classification space. Particularly, it is expected to be robust to different stochastic realizations of the trajectories (due to noise and other perturbation effects). Other metrics, measuring the time scales of convergence to the fixed point or focusing on specific properties of the trajectories are not expected to be robust for low signal-to-noise ratio (SNR) dynamics (see Figure 5). Such properties of the dynamics are typical to multi-unit recordings from neuronal networks. For example, in the benchmark set SNR is <3 and stimuli trajectories exhibit short timescales and do not necessarily converge to the fixed points. Rather, they only approach their vicinity.

To investigate the performance of recognition and classification using the hyperellipse metric we computed the similarity of various stimuli (17 stimuli from the benchmark set) with B1 mixture consisting of mono-molecular odorants: Benzaldehyde (S1); Benzyl Alcohol (S2); and Linalool (S3). This mixture was shown to include the dominant constituents of the Datura scent—an important flower nectar source of the moths—and moths stimulated with it elicit a “behavioral” response similar to stimulation by the full Datura scent. Similarly to B1, mixtures B2 and B3 elicit a behavioral response, while other 14 stimuli are not eliciting such a response and thereby marked “non-behavioral” (Riffell et al., 2013). The goal of the classification is to examine trajectories associated with all mixtures and determine which are similar to B1. Strong similarity indicates that the mixture is classified as “behavioral” as well. The goal of recognition is to decide instantaneously given a single trial whether the stimulus is B1. Notably, behavioral experiments show that similarity in responses to these different mixtures was not solely due to the concentration of the constituents making up the mixture, and therefore classification methods from neural dynamics are in need. For example, experiments show that when Linalool (component with the least concentration) is missing in B1 mixture, the new mixture becomes non-behavioral. Furthermore, adding other constituents from the Datura scent to replace Linalool in B1 mixture does not restore the ability of the mixture to elicit behavior.

**Figure 6:**
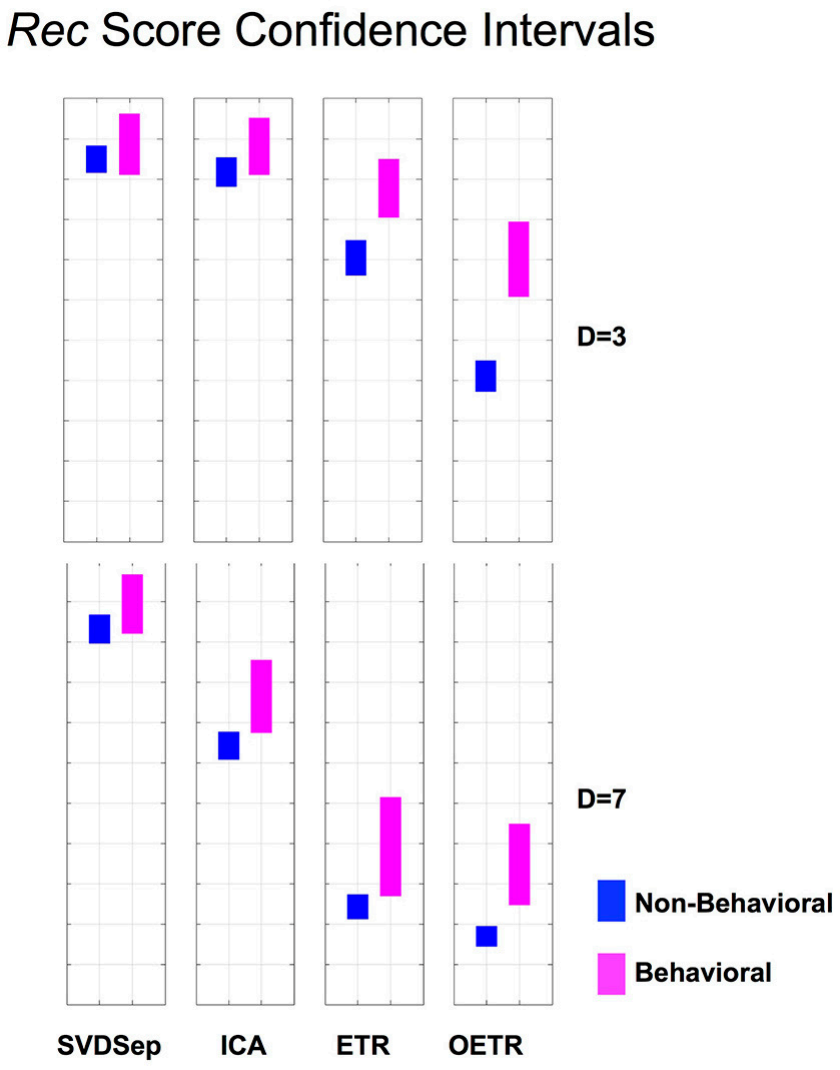
*Rec* score confidence intervals. Comparison of *Rec* confidence intervals (*P* = 0.01) for each of the methods for behavioral and non-behavioral classes. Two representative dimensions are shown: *D* = 3 (top) and *D* = 7 (bottom). For *D* = 3 the intervals belonging to ETR and OETR methods are separable. For *D* = 7 only the intervals belonging to the OETR method are separable. For other dimensions, the intervals exhibit similar separability features.

To compare between different approaches we constructed classification spaces using four methods: SVDSep, ICA, ETR, and OETR for the odorants S1–S8 (see table in Figure 2). We also varied the dimension *m* of the classification space from *m* = 1(*S*1) incrementally to *m* = 8(*S*1, …, *S*8). For each method and dimension of the classification space, we computed the score *Rec* for each trajectory (5 trials of 17 stimuli = 85 trajectories) with respect to B1 stimulus hyperellipse, which was chosen as a sphere with a fixed radius. We show our results in Figures 5, 6. To perform classification, *Rec* scores were computed for each dimension of the classification space with all stimuli and trials. The average 〈*Rec*〉 score was then computed over the trials of each stimulus. The distribution of average scores {〈*Rec*〉} for 17 stimuli is then normalized by the maximal *max*{〈*Rec*〉} value. A decision line for binary classification of “behavioral” (B class) or “non-behavioral” (S or E classes) is set as the midpoint between the mean of the distribution and maximal value: *d* = (〈{〈*Rec*〉}〉+1)/2 (depicted as blue line in the inset showing distributions for *m* = 7 in Figure 5). Values above *d* are classified as behavioral while values below *d* are classified as non-behavioral. In Figure 5 the bars that correspond to erroneous classification are marked with red color and correctly classified bars are marked with green color. We show the total accuracies of the four methods (precision × recall) for each dimension as a bar plot in the middle plot of Figure 5 and also show the distributions for *m* = 7 below it.

Classification results demonstrate that ETR and OETR are a significant improvement over other dimension reduction techniques, which in turn are a significant improvement over methods not employing dimension reduction. All methods perform poorly when the classification space is of *m* = 1 or *m* = 2 dimensions. Indeed, since B1 consists of three independent mono-molecular odorants, the results indicate that there is not enough data for discrimination based on fewer dimensions than the constituents of the tested stimulus. As the dimension increases, OETR achieves perfect accuracies for *m* ≥ 3. ETR achieves 100% accuracy for *m* = 3 and then stabilizes on the lower value of ≈70% with high precision but lower recall rates. ICA achieves 100% accuracy for *m* = 4 but when the dimension increases both precision and recall rates drop to values as for *m* = 1 and *m* = 2 dimensions. SVDSep produces constantly low accuracies for all dimensions. From the distributions, as shown for *m* = 7, we get insights on the differences in the performance of the methods. In particular, two stimuli are used for validation: B1, which is expected to produce a high *Rec* score, and E6 (control), which is expected to produce a low *Rec* score. We observe that the scores for B1 are high across methods, however in SVDSep B1 does not cross the threshold *d* line. For E6, only ETR and OETR successfully assign a significantly lower score to E6. As expected, individual odorants S1,…,S8 and non-behavioral mixtures E1,…E6 are far from threshold in OETR method and mostly far from threshold in ETR. The form of the distributions transforms from nearly uniform (for SVDSep) to spiked distribution with high values for B stimuli and low for other (for OETR). The latter form allows for the line *d* to easily separate the B class from other classes. Indeed we observe that while ETR is able to perform with high precision, it is unable to recall one of the behavioral stimuli (B2). Next, we proceed and test whether the methods are sensitive to the choice of the hyperellipse. In particular, we vary the radius of the B1 sphere in the range of 0.5–0.85 for dimension *m* = 8 and compute classification accuracies. We observe a clear separation between SVDSep/ICA and ETR/OETR groups of methods. We find that recall rates are stable across methods, however, precision rates are sensitive. Precision rates of SVDSep and ICA are less than 50% overall and indicate that these methods are not robust. By contrast, ETR and OETR are more robust. ETR achieves 100% precision for a range of 0.19 radii and OETR achieves 100% precision in a much wider range of 0.32.

To further quantify classification performance of different methods, we compute *Rec* score confidence intervals (99%) for binary classification of trajectories into behavioral or non-behavioral classes. We show representative results in Figure 6 for dimensions *D* = 3 and *D* = 7. Our results indicate that only the intervals that belong to the OETR method are consistently separable for all dimensions and thereby expected to support successful recognition. For other methods, the intervals appear to significantly overlap (SVDSep, ICA) or their boundaries are adjacent to each other (ETR).

For the recognition task, we compute the *Rec* score for *m* = 8 classification space for each trial (85 trials). If the score crosses a threshold of 70% of the *Rec* score of a target averaged trajectory (i.e., for more than 400 ms the trajectories are in the same hyperellipse) the tested trajectory is recognized as associated with the target stimulus. Using this method, we tested the recognition of the B1 hyperellipse (sphere of radius 0.65 in our case). As in classification, our results show that OETR is more accurate than other methods. Recall and precision rates of OETR and ETR are higher than the rates of SVDSep and ICA. Interestingly, when we test for the recognition of the B1 stimulus only (i.e., we expect to recognize only 5 noisy trajectories of B1 stimulus as positive) precision rates do not reach 100% in any of the methods; there are other trajectories marked positively as B1 although not B1. These trajectories turn out to be mainly from B class. We show that this is indeed the case by expanding the class of recognition and allowing for any stimulus in B class to be marked as B1. In such a case, we obtain 100% precision for OETR and ETR. These results demonstrate that noisy B trajectories appear extremely similar to each other in our recognition scheme. OETR is hence consistent with experimental observations and metrics which indicate that B class stimuli are behaviorally indistinguishable.

## 3. Conclusion

Classification of fixed point networks responses from sampled activity is a challenging fundamental problem, especially when the inputs into a network are highly variable and noisy. We have addressed this problem by proposing novel supervised classification methods, ETR and OETR, which identify a low-dimensional representation of a collection of matrices of multiple node time-series data from the network. Essentially, the ETR method combines dimension reduction and clustering of nodes to obtain a meaningful projection space for network responses. As a first step, the method employs dimension reduction based on matrix factorization to obtain response vectors for each stimulus. Next, it associates nodes to stimuli classes (clustering) and assigns a weight to each node within a class using its maximal response across stimuli. As a result, ETR orthogonalizes the response vectors, makes them more sparse, and provides a basis for the projection space. The OETR method is performed in addition to ETR to optimize the weights of each node in a cluster.

To test our methods we have established a benchmark database of multi channel time-series recordings from a real neural fixed point network: the antennal lobe in the *M. sexta* moth. We have shown that the OETR method allowed us to create a classification space that separates fixed points and their transient trajectories with high accuracy. Furthermore, it allowed us to represent and classify noisy mixtures of stimuli. In contrast, we have shown that traditional methods such as SVM and Boosting, that do not rely on dimension reduction, and classical matrix decomposition and reduction methods such as SVD and ICA, are not as successful as OETR at the classification of fixed point networks dynamics.

Recordings from the olfactory network are a valuable benchmark for testing classification. The network is known for its robustness in discrimination of scents composed of numerous distinct molecules in a highly dynamic environment. It has been established that the network employs fixed points for odor representation that are being read and processed by higher layer olfactory processing units (mushroom body in insects). In this respect, the ETR and OETR methods that we have proposed are simple to implement since they rely on a dominant mean pattern (first SVD mode) associated with each distinct odorant, maximum rule, and linear weights. Such components are natural to neural networks. Thereby application of proposed methods could help in understanding the processes that higher layer units employ to instantaneously read information from fixed point networks. While there has not been any explicit demonstration of selective ETR/OETR like coding in the *M. sexta* antennal lobe or downstream networks, such as the mushroom bodies, studies demonstrated that downstream neurons specifically respond to mixtures at the behaviorally effective ratios. The ratios of the constituent odorants were found critical for moth behavior and representation in the antennal lobe network (by altering the balance of excitation and inhibition; Kanzaki et al., 1991; Lei et al., 2013). In other words, downstream neurons only responded to the behaviorally effective mixture and at the proper ratio. This is the functionality that our classification and recognition methods are able to mimic.

Here we focused on fixed point networks. These networks are the most fundamental and simplest attractor networks. It is thereby plausible that our methodology will lay the foundations for future work to extend the methodology to other more complex attractor networks such as limit cycle networks, lines of fixed points networks, and ring attractor networks, which are ubiquitous networks in neural systems as well.

## 4. Methods and procedures

The benchmark data-set and code that implements the various methods are available online on GitHub: https://github.com/shlizee/fpnets-classification.

Below we include further information on the algorithms that we applied and compared with ETR and OETR methods.

### 4.1. SVM binary classification from raw data

In our application of SVM approach, a hyperplane *H* is created to divide the classes into their proper labels (−1 or 1). The hyperplane is designed to give the largest margin between these classes. It is constructed as $w^{T}x_{i}+b=0$, where *w* is the vector of weights for the features in *x*, and $\frac{1}{∥w∥}$ is the distance to *H*_+_ and *H*_−_. In order to obtain the optimal hyperplane, $\frac{1}{∥w∥}$ must be maximized, or ∥*w*∥ minimized. Thus the Maximum Likelihood Estimator (MLE) for SVMs is found by minimizing $\frac{1}{N}\overset{N}{\underset{i=1}{\sum }}max\left\{0,1-y_{i}w^{T}x_{i}\right\}+\lambda ∥w{∥}^{2}$, where *y* is the class label, and λ is the penalty on the weights *w*.

### 4.2. Class imbalances

When the error or misclassification percentage is the only metric taken into account during training, learning algorithms of SVM type appear to work well. However, during testing, these methods could produce inaccurate results. The reason for the discrepancy stems from the imbalanced representation of classes in the data. For example, in the case of two classes, there is a possibility that the first class would not be classified at all, while the second class would be perfectly classified since there are significantly more data points in the second class. Therefore, different approaches for working with imbalanced data are required. One approach is to change the performance metric. Typically, two informative measures are *precision* (*P*) and *recall* (*R*), defined as $P=\frac{tp}{tp+fp},R=\frac{tp}{tp+fn}$, where *tp* is the number of true positives, *fp* is the number of false positives, and *fn* is the number of false negatives. While using these metrics did not improve the performance of SVM on our benchmark, we do employ the *P* and *R* metrics to quantify and compare the performance of ETR and OETR with other methods. An alternate approach is to resample the data-set. This includes over-sampling classes with fewer instances or under-sampling the larger classes to achieve equal balance. In practice, however, these strategies often cause biases such as overfitting (in the case of over-sampling) or loss of information (in the case of under-sampling). Another technique to improve imbalanced classification is boosting. Boosting can improve the performance of weak classifiers, such as decision trees, by resampling the training data using assigned weights. Boosting techniques constantly change weak learning directions until they converge toward a strong learner (see the following subsection for an example of boosting technique that we applied).

### 4.3. RUSBoost algorithm

Here we applied the RUSBoost algorithm to demonstrate that treatment of class imbalances is insufficient for fixed point responses in the benchmark that we have collected. RUSBoost is a hybrid method for random under-sampling and boosting designed to improve performance of imbalanced data classification. The algorithm randomly removes instances of majority classes to try to equalize them with minority classes. RUSBoost has been shown to work well, as well as SMOTEBoost, and perform simply and speedily (Seiffert et al., 2010). RUSBoost algorithm works as follows: Given a training set *S* of examples (*x*_1_, *y*_1_)…(*x*_*n*_, *y*_*n*_) with a minority class *y*^τ^ ∈ *Y*, |*Y*| = 2, a new set *D*′ is generated using random under-sampling, and a weak learner (i.e., a decision tree) is called to create a hypothesis *h*_*t*_. The pseudo loss, for *S* and *D*_*t*_, is then calculated using $\epsilon_{t}=\underset{\left(i,y\right):y_{i}\neq y}{\sum }D_{t}\left(i\right)\left(1-h_{t}\left(x_{i},y_{i}\right)+h_{t}\left(x_{i},y_{i}\right)\right)$. The weight update parameter is then derived with: $\alpha_{t}=\frac{\epsilon_{t}}{1-\epsilon_{t}}$. Normalization yields the final hypothesis $H\left(x\right)=argmax_{y\in Y}\overset{T}{\underset{t=1}{\sum }}h_{t}\left(x,y\right)log\frac{1}{\alpha_{t}}$.

### 4.4. ICA

We are comparing our methods with the Infomax ICA algorithm, which for given observed components (in our case the matrix *L*^*T*^) finds a linear transformation *A* from *L*^*T*^ to *S*, i.e., *S* = *AL*^*T*^. The matrix *S* is decomposed into independent components that minimize mutual information. The assumption of the ICA model is that the components in *L* are statistically independent. We are using the implementation of the Infomax ICA based on Bell and Sejnowski (1995), in which the maximum likelihood estimation is found by using the log-likelihood in the form of $LH=\overset{T}{\underset{t=1}{\sum }}\overset{n}{\underset{i=1}{\sum }}logf_{i}\left(w_{i}^{T}S\left(t\right)\right)+Tlog|det\left(A\right)|$ where *f*_*i*_ is the density functions of the columns of *L*^*T*^. This is equivalent to the Infomax Principle, $M_{2}=H\left(\phi_{1}\left(w_{1}^{T}x\right),\ldots ,\phi_{n}\left(w_{n}^{T}x\right)\right)$ where ϕ_*i*_ are non-linear scalar functions. We used Python software package that implements the Infomax algorithm (Higuera et al., 2016).

## Author contributions

Inception, validation. manuscript revision and funding: ESh and JR. Experimentation and manuscript writing: ESh, DB, and JR. Computational research and Implementation: DB and ESh. Data acquisition: ESa and JR. Data processing: ESa, DB, and ESh.

### Conflict of interest statement

The authors declare that the research was conducted in the absence of any commercial or financial relationships that could be construed as a potential conflict of interest.
